# Supplementary material for: The Human Chk1 Inhibitor CHIR-124 Shows Multistage Activity against the Human Malaria Parasite Plasmodium falciparum via Polypharmacological Inhibition of PfArk1 and Hemozoin Formation
Source: ACS Chem Biol. 2026 Jun 13;21(7):1738–48. doi: 10.1021/acschembio.6c00264 (PMC13386468; doi:10.1021/acschembio.6c00264)
Supplement: Supplementary file 1 [file cb6c00264_si_001.pdf]

SUPPORTING INFORMATION

The Human Chk1 Inhibitor CHIR-124 Shows Multistage Activity Against the Human Malaria Parasite *Plasmodium falciparum* via Polypharmacological Inhibition of *Pf*Ark1 and Hemozoin Formation

Kathryn J. Wicht,*[1][2][3] John G. Woodland,[1][2][3] Larnelle F. Garnie,[3] Henrico Langeveld,[4][5], Dale Taylor,[1] Luiz C. Godoy,[6] Charisse Flerida A. Pasaje,[6] Mariana Laureano de Souza,[7] Jair L. Siqueira-Neto,[7] Sonja Ghidelli-Disse,[8] Maria Jose Lafuente-Monasterio,[9] Francisco-Javier Gamo,[9] Dina Coertzen,[4] Janette Reader,[4] Mariëtte van der Watt,[4] Jessica L. Bridgford,[10] Gareth Girling,[11] Rachael Coyle,[11][12] Christian Scheurer,[13][14] Sergio Wittlin,[13][14] Arne Alder,[15][16][17] Tim-Wolf Gilberger,[15][16][17] Marcus C.S. Lee,[11][12] Till S. Voss,[13][14] Elizabeth A. Winzeler,[7] David A. Fidock,[10] Jacquin C. Niles,[6] Lyn-Marié Birkholtz,[4][5] Lauren B. Coulson,[1] and Kelly Chibale*[1][2][3]

[1] Holistic Drug Discovery and Development (H3D) Centre, University of Cape Town, Rondebosch 7701, South Africa.

[2] South African Medical Research Council Drug Discovery and Development Research Unit, Department of Chemistry and Institute of Infectious Disease and Molecular Medicine, University of Cape Town, Observatory 7925, South Africa.

[3] Department of Chemistry, University of Cape Town, Rondebosch 7701, South Africa.

[4] Department of Biochemistry, Genetics and Microbiology, Institute for Sustainable Malaria Control, University of Pretoria, Hatfield 0028, South Africa.

[5] Department of Biochemistry, Stellenbosch University, Stellenbosch 7600, South Africa.

[6] Department of Biological Engineering, Massachusetts Institute of Technology, Cambridge, MA 02139, USA.

[7] Department of Pediatrics and Skaggs School of Pharmacy and Pharmaceutical Sciences, University of California, San Diego, La Jolla, CA 92093-0657, USA.

[8] Cellzome GmbH, a GSK Company, 69117 Heidelberg, Germany.

[9] GlaxoSmithKline, Tres Cantos Medicines Development Campus, Madrid 28760, Spain.

[10] Department of Microbiology and Immunology, and Center for Malaria Therapeutics and Antimicrobial Resistance, Division of Infectious Diseases, Department of Medicine, Columbia University Irving Medical Center, New York, NY 10032, USA.

[11] Wellcome Sanger Institute, Wellcome Genome Campus, Hinxton, Cambridge, CB10 1SA, UK.

[12] Division of Biological Chemistry and Drug Discovery, Wellcome Centre for Anti-Infectives Research, University of Dundee, Dundee, DD1 5EH, UK.

[13] Department of Medical Parasitology and Infection Biology, Swiss Tropical and Public Health Institute, 4123 Allschwil, Switzerland.

[14] University of Basel, 4001 Basel, Switzerland.

[15] Centre for Structural Systems Biology, 22607 Hamburg, Germany

[16] Bernhard Nocht Institute for Tropical Medicine, Department of Cellular Parasitology, 20359 Hamburg, Germany

[17] University of Hamburg, Department of Biology, 20146 Hamburg, Germany

***Corresponding authors:** Kelly Chibale; Orcid.org/0000-0002-1327-4727; kelly.chibale@uct.ac.za; Kathryn J. Wicht, Orcid.org/0000-0001-6145-9956, kathryn.wicht@uct.ac.za

CONTENTS

1. Supplementary Figures

Supplementary Figure S1 – Competitive inhibition data from Kinobeads assay
Supplementary Figure S2 – Cross-resistance with Southeast Asian lines
Supplementary Figure S3 – Cross-resistance with DNA-barcoded resistant mutant lines
Supplementary Figure S4 – Conditional knockdown (cKD) studies
Supplementary Figure S5 – Biochemical *Plasmodium* kinase activities
Supplementary Figure S6 – Cellular heme fractionation data
Supplementary Figure S7 – Metal chelation studies
Supplementary Figure S8 – Resistance selection profiling

2. Supplementary Tables

Supplementary Table S1 – Kinobead MS/MS Data
Supplementary Table S2 – IC_{50} data for asexual blood stage activities
Supplementary Table S3 – Liver stage activity and cytotoxicity data
Supplementary Table S4 – List of barcoded lines in the AReBar cross-resistance screening pool
Supplementary Table S5 – Conditional knockdown (cKD) data
Supplementary Table S6 – List of oligonucleotides for donor vector construction

3. Methods

3.1 Kinobead screening

3.2 *Plasmodium falciparum* parasitology methods
3.2.1 Parasite culture
3.2.2 Antiplasmodial activity assessments
3.2.3 Stage specificity assay
3.2.4 Stage-specific gametocyte production and luciferase assay
3.2.5 Liver stage assay
3.2.6 Liver stage cytotoxicity
3.2.7 Conditional knockdown assays
3.2.8 Cross-resistance profiling using the Antimalarial Resistome Barcode sequencing assay
3.2.9 Combination studies via fixed-ratio isobolograms
3.2.10 Rate- and stage-specific morphological evaluations and inhibitor effect reversibility

3.3 β-hematin and hemozoin formation inhibition assays and metal chelation studies
3.3.1 NP-40 based extracellular β-hematin inhibition assay
3.3.2 Cellular heme fractionation assay
3.3.3 Metal chelation studies

3.4 Biochemical assays
3.4.1 KinaseSeeker™ assay

1. Supplementary Figures

Fig. S1

A

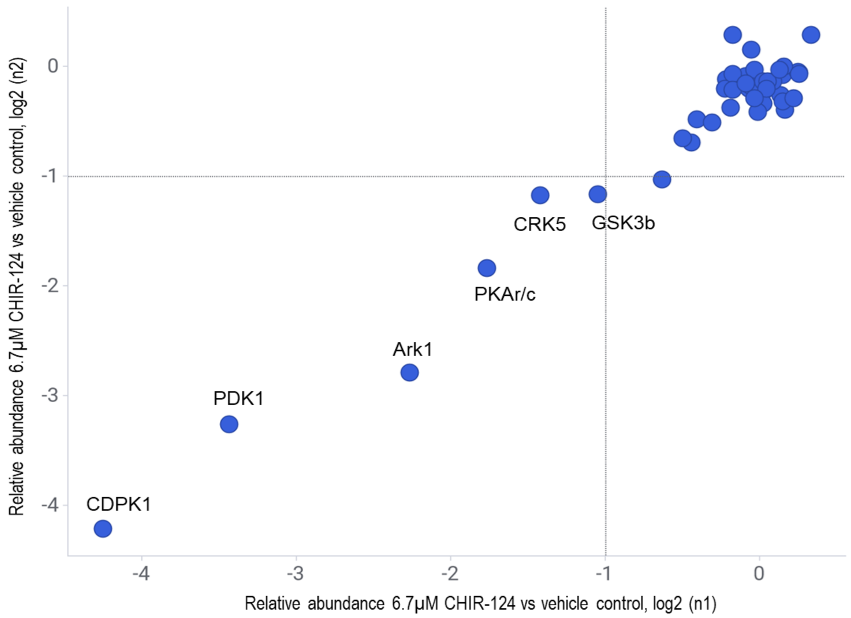

B

Gene name	UniProt ID	Protein description	Former name	IC_{50} (μM) n=2	K_d^{app} (μM)
PF3D7_0217500	P62344	CDPK1, Calcium-dependent protein kinase 1	PFB0815W	0.0823*	0.04
				0.0823*	0.04
PF3D7_1121900	Q8IIE7	PDK1, Serine/threonine protein kinase, putative	PF11_0227	0.21	0.12
				0.15	0.08
PF3D7_0605300	C6KSQ1	Ark1, Serine/threonine protein kinase	PFF0260W	0.32	0.18
				0.28	0.16
PF3D7_1223100	Q7KQK0	PKAr, CAMP-dependent protein kinase regulatory subunit, putative	PFL1110C	0.83	0.64
				0.87	0.61
PF3D7_0934800	Q7KQA0	PKAc, cAMP-dependent protein kinase catalytic subunit	PFI1685W	0.88	0.63
				0.85	0.59
PF3D7_0615500	C6KSZ6	CRK5, Cdc2-related protein kinase 5	PFF0750W	1.7	1.04
				2.9	1.89
PF3D7_0312400	O77344	GSK3beta, Glycogen synthase kinase 3	PFC0525C	2.8	1.23
				3.1	1.32
PF3D7_0805700	C0H4R8	FIKK8, Serine/threonine protein kinase, FIKK family	MAL8P1.203	8.2	7.87
				3.5	2.97

*0.0823 μM was the lowest compound concentration used.

Supplementary Figure S1. Kinobeads profiling of CHIR-124 was conducted using a dose-dependent competition assay (starting at 20 μM, followed by serial 1:3 dilutions). (**A**) Schematic representation of two independent experiments. (**B**) Summary table detailing IC_{50} values and apparent K_d values, which reflect the binding affinity of the protein to the compound immobilized on the beads. Data are reported for the seven competed kinases, including the regulatory subunit of PKAr/c.

Fig. S2

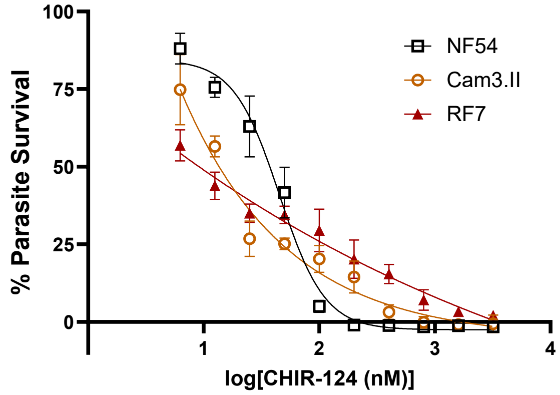

Supplementary Figure S2. Cross resistance with Southeast Asian strains. CHIR-124 was profiled against the *P. falciparum* lines Cam3.II (resistant to chloroquine and artemisinin) and RF7 (resistant to artemisinin and piperaquine) and displayed more shallow dose response curves.

Fig. S3

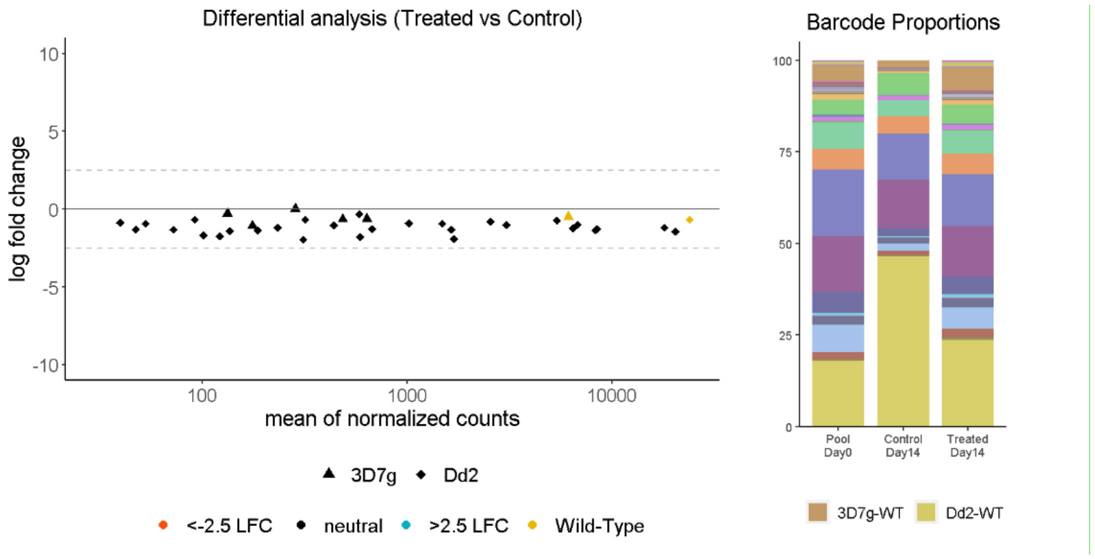

Supplementary Figure S3. Cross resistance profiling using the Antimalarial Resistome Barcode sequencing (AReBar) assay. A pool of 45 DNA-barcoded resistant mutant lines (**Supplementary Table S4**) were exposed to 3×IC_{50} concentrations of CHIR-124. No mutant strain enrichment was observed after 14 days. *Left panel*, differential analysis comparing CHIR-124-treated parasites versus untreated control pool, with the log2 fold change (LFC) of individual barcoded lines (y-axis) and barcode counts (x-axis) shown. Dotted lines indicate ±2.5 LFC relative to untreated control lines, and symbols indicate strain background of mutant lines. *Right panel*, barcode proportions of each line at day 0, and day 14 for the untreated control and CHIR-124-treated pool.

Fig. S4

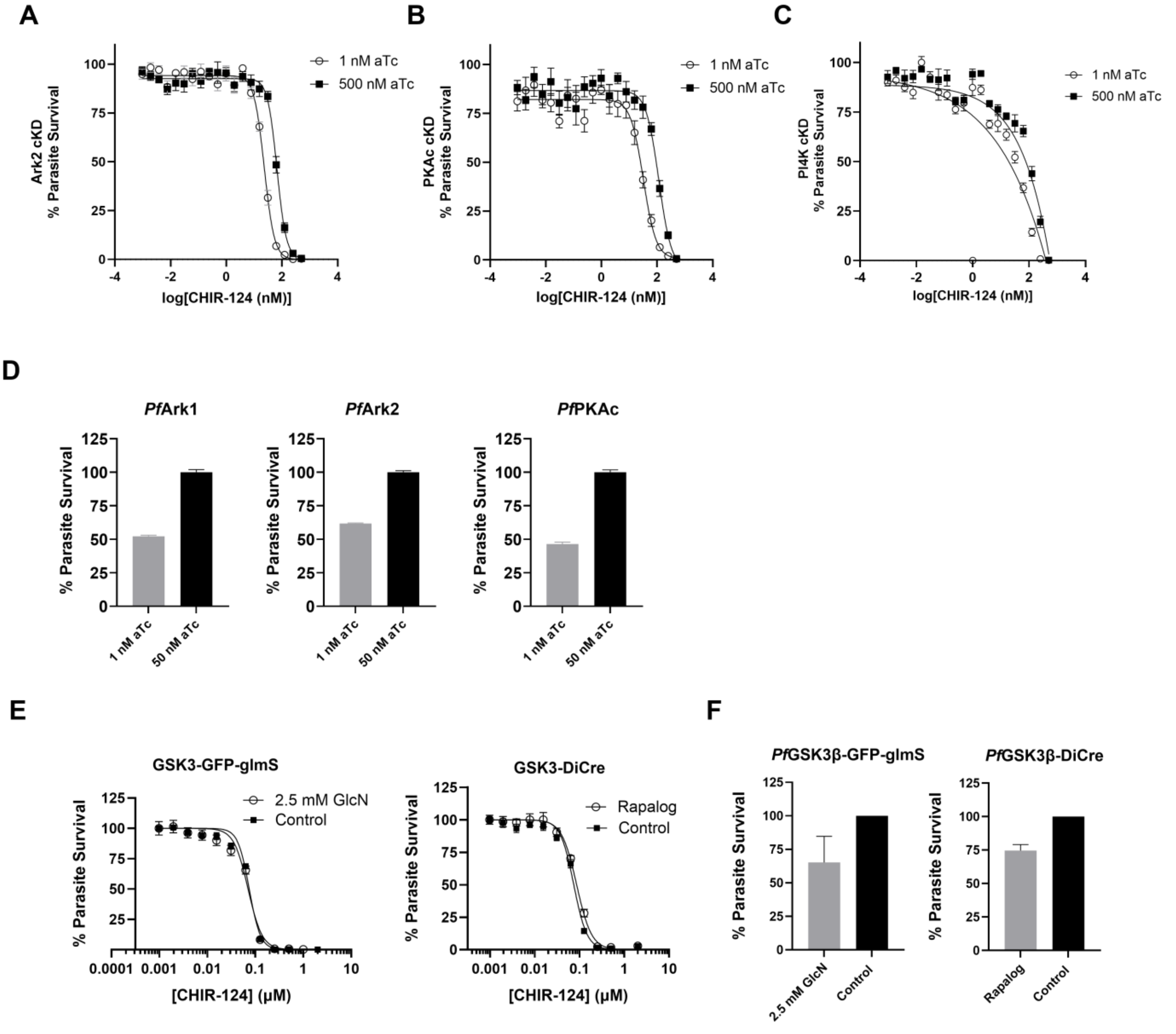

Supplementary Figure S4. Conditional knockdown (cKD) studies with CHIR-124. TetR-based anhydrotetracycline (aTc)-controlled conditional knockdown (cKD) studies with the (**A**) *Pf*Ark2 cKD line, (**B**) *Pf*PKAc cKD line and (**C**) *Pf*PI4K cKD line whereby expression of the kinase was regulated by the concentration of aTc on the culture during the 72 h assay. The *Pf*PI4K cKD represents the negative control demonstrating that the small (2–3 fold) curve shift to the left for the culture under 1 nM aTc (grey), relative to the 500 nM aTc line where the kinase was fully expressed, indicates a negligible interaction between CHIR-124 and the specified kinase targets. Data represents mean ± SEM where N,n = 3,3. (**D**) Parasite viability for untreated cKD lines used in this study represented by 72 h growth assays in presence of high (50 nM, WT condition) and low (1 nM, knockdown) aTc. Data represents mean ± SEM where N,n = 3,3. Viability data for the *Pf*PI4K cKD has been previously reported.[60] (**E**) CHIR-124 tested against inducible lines *Pf*GSK3β-GFP-glmS and *Pf*GSK3β-DiCre comparing 2.5 mM glucosamine (GlcN) or 250 nM rapalog versus untreated control respectively. No difference in drug sensitivity was observed in a 96 h proliferation assay indicating that *Pf*GSK3β is unlikely to contribute to the mode of action of CHIR-124. Data represents mean ± SEM where N,n = 3,2). (**F**) Parasite viability for untreated *Pf*GSK3β-GFP-glmS or *Pf*GSK3β-DiCre used in this study represented by 96 h growth assays normalized to the control (100% survival) in the presence of 2.5 mM GlcN or rapalog, respectively. Data represents mean ± SEM where N = 3-4.

Fig. S5

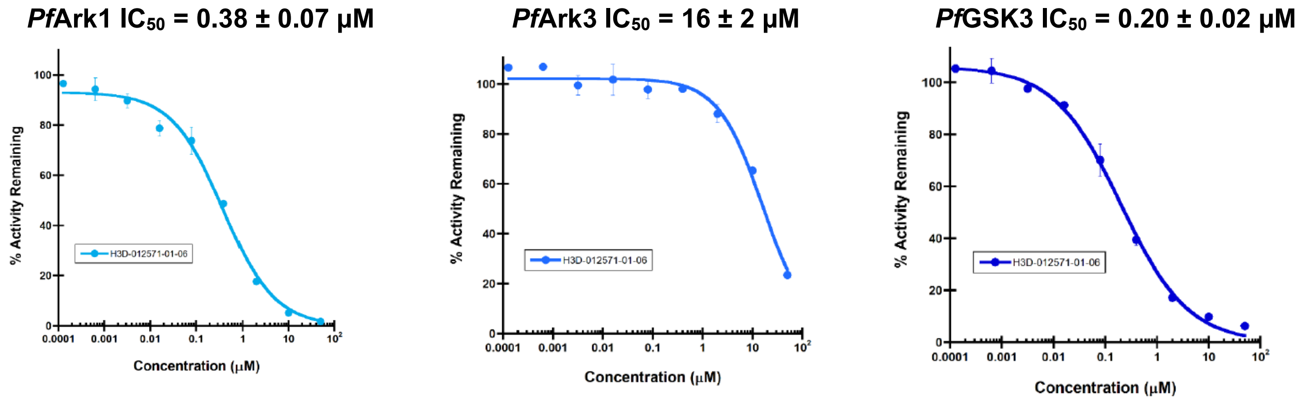

Supplementary Figure S5. *Plasmodium* kinase biochemical activities against *Pf*Ark1, *Pf*Ark3 and *Pf*GSK3β. Mean ± SD shown above their dose-response curves with error bars representing SD from screening against the specified kinase in duplicate.

Fig. S6

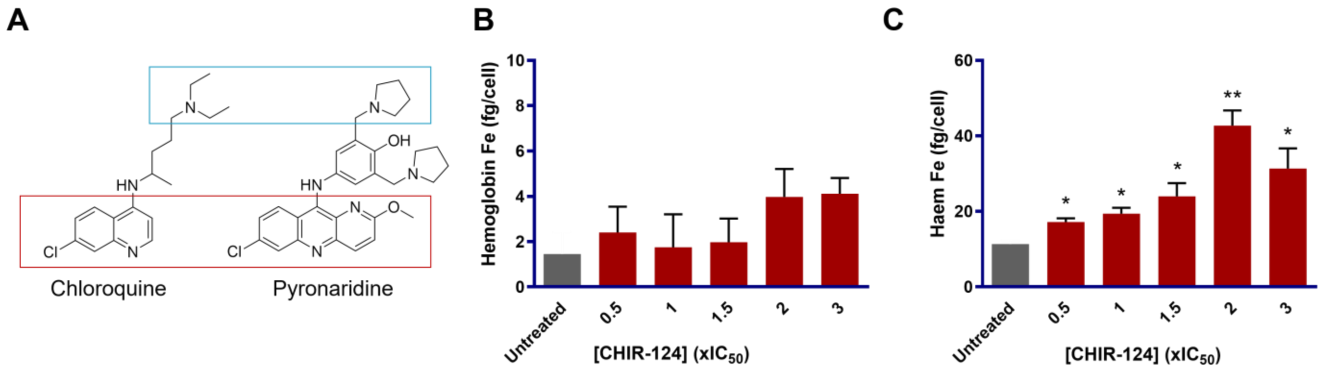

Supplementary Figure S6: (**A**) Validated clinical hemozoin formation inhibitors, chloroquine and pyronaridine, highlighting the typical pharmacophore for this class of inhibitor which contains planar heteroaromatics (red box) and basic centers (blue box). Cellular heme fractionation assays with multiples of the CHIR-124 IC_{50} showing heme Fe (fg/cell) levels from the (**B**) free hemoglobin fraction, and (**C**) absolute free heme fraction.

Fig. S7

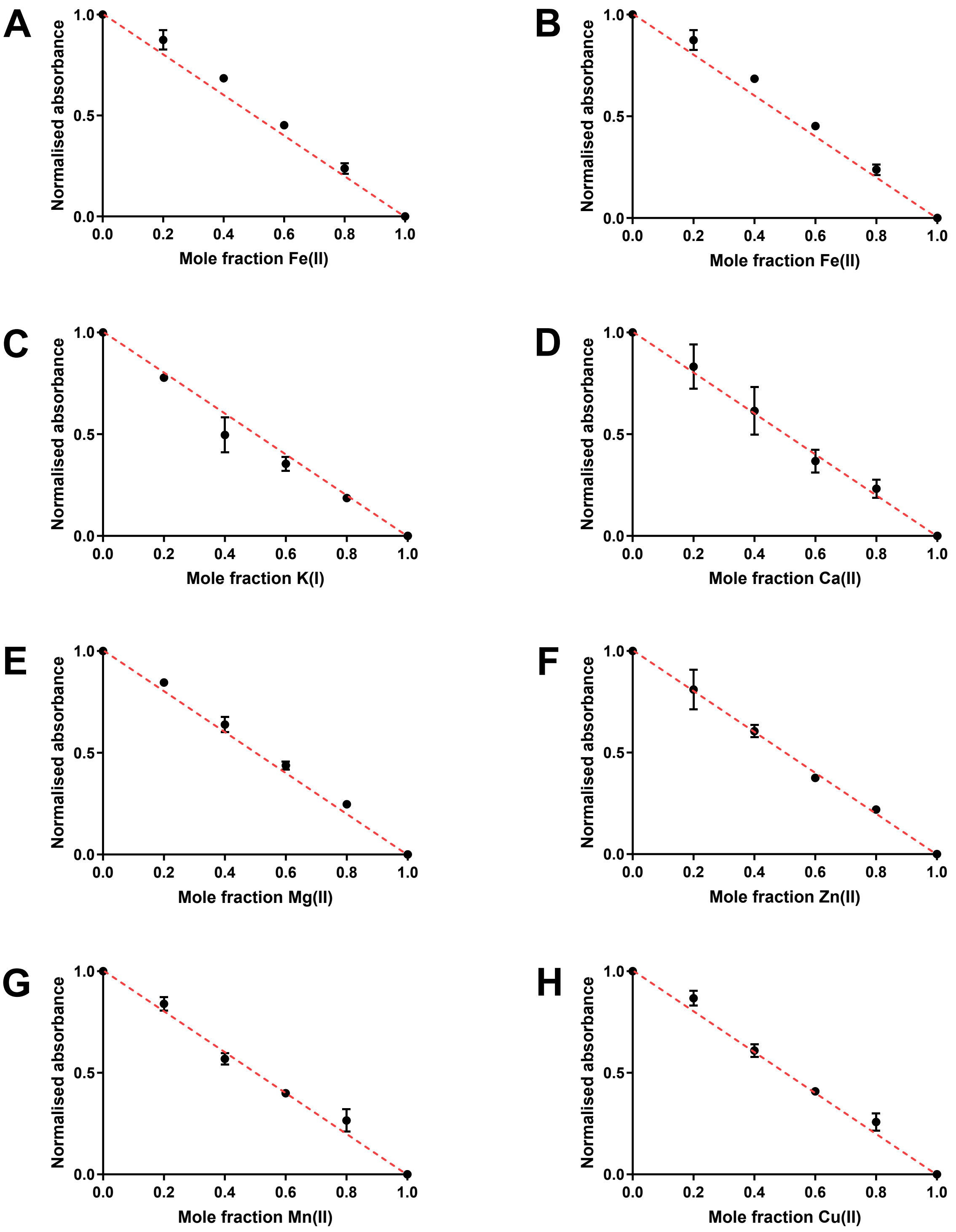

Supplementary Figure S7. Metal chelation studies via spectrophotometric titrations of CHIR-124 with (**A**) Fe(**II**), (**B**) Fe(**III**), (**C**) K(I), (**D**) Ca(**II**), (**E**) Mg(**II**), (**F**) Zn(**II**), (**G**) Mn(**II**) and (**H**) Cu(**II**) showing, in all cases, a very weak capacity for chelation of these metal ions with CHIR-124. Dashed red lines indicate Beer-Lambert law dilutions curves; deviation from that line indicates likely chelation. Error bars represent technical duplicates of each experiment.

Fig. S8

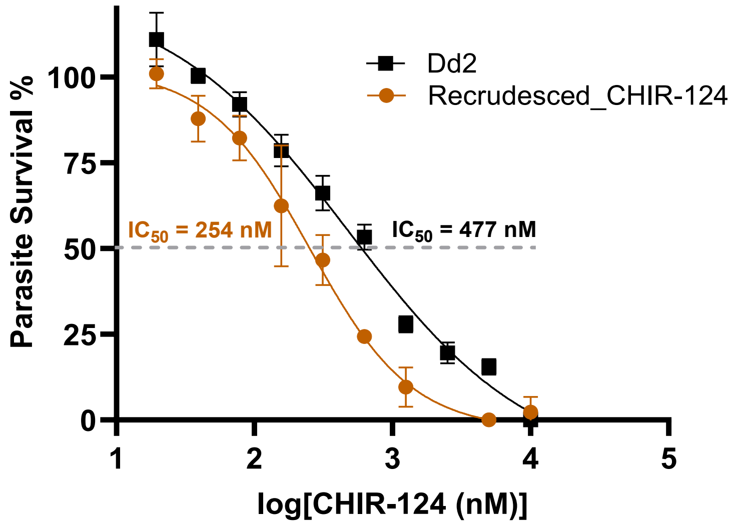

Supplementary Figure S8: Resistance selection profiling. Parasites recrudesced while the flask was being cultured without CHIR-124 pressure on day 49. The parasites were subjected to a pLDH assay on one occasion whereupon they were found to be approximately 2-fold more sensitive to CHIR-124 and subsequently put back on CHIR-124 pressure at 5×IC_{50} without further profiling. Data points and error bars represent mean ± SD from one biological repeat.

2. Supplementary Tables

Supplementary Table S1. Kinobeads MS/MS Data**.** *See Excel file.* MS/MS data for profiling of CHIR-124 (GSK3210608, CAS No. 405168-58-3) on Kinobeads in *P. falciparum* extracts: 10 samples were analyzed in parallel (TMT 10-plex) to generate values for the affinity of the beads to the bound proteins (“depletion” values, 4 samples) and to generate IC_{50} values (6 samples) in a single experiment. Samples 1 and 2 represent the vehicle control. Samples 3 and 4 were done in the same way, but while the beads were discarded after the first incubation step the extract was incubated with fresh beads to measure how much protein could rebind to the fresh beads (protein was depleted from the extract by first bead-binding). Apparent dissociation constants were determined by taking into account the protein depletion by the Kinobeads. Samples 5–10 were used to generate IC_{50} values by adding compound over a range of concentrations (20 µM, 1:3 dilutions).

Experiment number	Compound immobilized on beads	"Free" compound added to extract for competition	Sample description	Compound Concentration (µM)
X048537	Kinobeads	GSK3210608 CHIR-124	Vehicle control (n=1)	0
			Vehicle control (n=2)	0
			Re-binding of NBF to fresh beads (n=1)	0
			Re-binding of NBF to fresh beads (n=2)	0
			Dose response competition over 6 concentrations	20.0
				6.67
				2.22
				0.74
				0.25
				0.08
X048538	Kinobeads	GSK3210608 CHIR-124	Vehicle control (n=1)	0
			Vehicle control (n=2)	0
			Re-binding of NBF to fresh beads (n=1)	0
			Re-binding of NBF to fresh beads (n=2)	0
			Dose response competition over 6 concentrations	20.0
				6.67
				2.22
				0.74
				0.25
				0.08

Supplementary Table S2. IC_{50} data for asexual blood stage activities. Inhibition of the WT drug-sensitive (*Pf*NF54) and drug-resistant strains by CHIR-124, showing the mean IC_{50} ± SEM and Hill slope calculated from N,n = 2-3,2 repeats.

Strain	Assay detection	Mean IC_{50} ± SEM (μM)	Mean Hill slope
*Pf*NF54 (WT)	pLDH	0.14 ± 0.03	-2.9 ± 0.4
*Pf*Dd2	pLDH	0.44 ± 0.05	-1.3 ± 0.5
*Pf*K1	pLDH	0.14 ± 0.03	-1.1 ± 0.2
*Pf*NF54 (WT)	SYBR Green I	0.033 ± 0.002	-2.2 ± 0.2
*Pf*Cam3.II	SYBR Green I	0.016 ± 0.002	-0.5 ± 0.3
*Pf*RF7	SYBR Green I	0.010 ± 0.001	-0.1 ± 0.7

Supplementary Table S3. Liver stage activity and cytotoxicity data.

	Liver cytotoxicity HepG2 IC_{50} (μM)	Liver-stage PbLuc IC_{50} (μM)
Rep 1	5.4	0.30
Rep 2	4.3	0.34
Rep 3	3.3	0.30
**Mean**	**4.3**	**0.31**
**SEM**	**0.6**	**0.01**

Supplementary Table S4. List of barcoded lines in the AReBar cross-resistance screening pool. The line name, indicating strain background (*Pf*3D7 or *Pf*Dd2) and mutation are shown in column 1. Gene description, gene ID, and proportion of each line in the pool at day 0 and after 14 days of CHIR-124 treatment (3×IC_{50}) are shown.

Line name	Gene Description	Gene ID	Input Day 0 (%)	CHIR-124 Day 14 (%)
3D7-WT	Wild type		4.31	6.43
3D7 ABCI3 R2180P	ABC transporter I family member 1	PF3D7_0319700	0	0.02
3D7 ACS10 M300I	Acyl CoA synthase	PF3D7_0525100	0.39	0.48
3D7 ACS11 D648Y	Acyl CoA synthase	PF3D7_1238800	0	0
3D7 ACS11 E668K	Acyl CoA synthase	PF3D7_1238800	0.01	0
3D7 ATP2 CNV2	Phospholipid-transporting ATP2	PF3D7_1219600	0.01	0.03
3D7 DHFR-TS G378E	Dihydrofolate reductase-thymidylate synthase	PF3D7_0417200	0.16	0.14
3D7 DHFR-TS I403L	Dihydrofolate reductase-thymidylate synthase	PF3D7_0417200	0.17	0.37
3D7 FTb A515T	Farnesyltransferase subunit beta	PF3D7_1147500	0.49	0.63
3D7 MDR2 K840N	Multidrug resistance protein 2	PF3D7_1447900	0.03	0.02
3D7 NCR1 A1108T	Niemann-Pick type C1-related protein	PF3D7_0107500	0.09	0.15
Dd2-WT	Wild type		18.08	23.58
Dd2 AcAS A597V	Acetyl-CoA synthetase	PF3D7_0627800	0.12	0.07
Dd2 ATP4 G358S	Non-SERCA-type Ca2+ -transporting P-ATPase (ATP4)	PF3D7_1211900	1.62	0.91
Dd2 ATP4 A353E CARL I1139K	ATP4+CARL	PF3D7_1211900	0.09	0.06
Dd2 CARL I1139K	Cyclic amine resistance locus (CARL)	PF3D7_0321900	0.16	0.13
Dd2 CARL L1073Q	Cyclic amine resistance locus (CARL)	PF3D7_0321900	0.07	0.09

Dd2 CARL V1103L	Cyclic amine resistance locus (CARL)	PF3D7_0321900	0.13	0.09
Dd2 CPSF Y408S (edit)	Cleavage and polyadenylation specific factor	PF3D7_1438500	0.01	0.01
Dd2 CPSF Y408S (sel)	Cleavage and polyadenylation specific factor	PF3D7_0709000	0	0
Dd2 CRT M343L	Chloroquine resistance transporter	PF3D7_1250200	0.59	0.5
Dd2 CSC1 L800P	CSC1-like protein putative	PF3D7_MIT02300	0.25	0.31
Dd2 cytBC1 G33V	Cytochrome b	PF3D7_MIT02300	0.21	0.17
Dd2 cytBC1 V284L	Cytochrome b	PF3D7_0417200	0.32	0.36
Dd2 DHFR-TS S216R	Dihydrofolate reductase-thymidylate synthase	PF3D7_0603300	1.43	1.2
Dd2 DHODH C276Y	Dihydroorotate dehydrogenase	PF3D7_0603300	4.01	5.14
Dd2 DHODH F227I	Dihydroorotate dehydrogenase	PF3D7_0603300	0.02	0.01
Dd2 DHODH I263F	Dihydroorotate dehydrogenase	PF3D7_1451100	0.02	0.01
Dd2 DHODH L531F	Dihydroorotate dehydrogenase	PF3D7_1451100	0.05	0.04
Dd2-pol-delta D308A-E310A	DNA polymerase delta	PF3D7_1017000	0.56	0.34
Dd2 eEF2 L755F	Elongation factor 2	PF3D7_1128400	1.18	1.3
Dd2 eEF2 Y186N	Elongation factor 2	PF3D7_1332900	0.03	0.03
Dd2 GGPPS S228T	Geranylgeranyl diphosphate synthase	PF3D7_1332900	0.31	0.16
Dd2 IleRS E180D	Ile-tRNA synthetase	PF3D7_1332900	7.15	6.25
Dd2 IleRS L810F	Ile-tRNA synthetase	PF3D7_1343700	5.43	5.75
Dd2 IleRS V500A	Ile-tRNA synthetase	PF3D7_1343700	17.98	14.06
Dd2 kelch13 C580C	Kelch protein K13	PF3D7_1343700	15.16	13.94
Dd2 kelch13 C580Y	Kelch protein K13	PF3D7_0908800	6.36	4.72
Dd2 kelch13 R539T	Kelch protein K13	PF3D7_0523000	0.81	0.89
Dd2 MDR1 F1072L	Multidrug resistance protein 1	PF3D7_0509800	0.07	0.05
Dd2 PI4K CNV	Phosphatidylinositol 4-kinase	PF3D7_0509800	2.48	2.59
Dd2 PI4K S1320L+L1418F	Phosphatidylinositol 4-kinase	PF3D7_0509800	7.22	5.9
Dd2 PI4K S743F+H1484Y	Phosphatidylinositol 4-kinase	PF3D7_1213800	1.92	2.37
Dd2 ProRS L482H	Pro-tRNA synthetase	PF3D7_1011400	0.42	0.66
Dd2 UDP-GT F37V	UDP-galactose transporter	PF3D7_1017000	0.05	0.03

Supplementary Table S5. Conditional knockdown (cKD) data.

**cKD Line[a]**		Reduced expression (–Shield-1) IC_{50} (nM)	Full expression (+Shield-1) IC_{50} (nM)
*Pf*PDK1	Rep1	77.1	63.7
	Rep2	58.8	58.1
	Rep3	60.4	73.4
	**Mean IC_{50} ± SEM**	**65 ± 8**	**65 ± 6**
*Pf*NF45 control	Rep1	28.2	24.7
	Rep2	45.0	19.2
	Rep3	34.3	33.4
	**Mean IC_{50} ± SEM**	**36 ± 7**	**26 ± 6**
**cKD Line[b]**		Reduced expression (1 nM aTc) IC_{50} (nM)	Full expression (500 nM aTc) IC_{50} (nM)
*Pf*Ark1	Rep1	2.4	77.0
	Rep2	2.8	54.3
	Rep3	2.4	61.7
	**Mean IC_{50} ± SEM**	**2.5 ± 0.2**	**64 ± 9**
*Pf*Ark2	Rep1	26.6	76.1
	Rep2	22.1	65.0
	Rep3	21.1	58.8
	**Mean IC_{50} ± SEM**	**23 ± 2**	**67 ± 7**
*Pf*PKAc	Rep1	32.0	99.7
	Rep2	24.8	102.0
	Rep3	21.4	85.4
	**Mean IC_{50} ± SEM**	**26 ± 4**	**95 ± 7**
*Pf*PI4K	Rep1	49.1	129.0
	Rep2	59.2	92.0
	**Mean IC_{50} ± SEM**	**54 ± 7**	**110 ± 26**
**cKD Line[c]**		Reduced expression (2.5 mM GlcN) IC_{50} (nM)	Full expression (PBS vehicle) IC_{50} (nM)
GSK3β-GFP-glmS	Rep1	70.9	74.5
	Rep2	70.3	74.7
	Rep3	70.4	74.6
	**Mean IC_{50} ± SEM**	**70.5 ± 0.2**	**74.58 ± 0.04**
**cKD Line[d]**		Reduced expression (250 nM rapalog) IC_{50} (nM)	Full expression (RPMI medium) IC_{50} (nM)
GSK3β-DiCre	Rep1	85.5	75.4
	Rep2	86.8	73.7
	Rep3	86.7	74.2
	**Mean IC_{50} ± SEM**	**86.3 ± 0.4**	**74.5 ± 0.5**

[a] Using the method by Hitz *et al.*[61]
[b] Using the method by Goldfless *et al.*[62]
[c] Using the method by Burda et al.[63]
[d] Using the method by Jones et al.[64]

Supplementary Table S6. List of oligonucleotides for donor vector construction.

Description	Nucleotide sequence
Ark1 RHR forward sequence	aacccggaattcgagctcggGGTTTCACAACAAATGACATA
Ark1 RHR reverse sequence	cgagagattgggtattagacctagggataacagggtaatGAAGATGAGTTTAGTGAGGATA
Ark1 sgRNA target site	TCTTACGCGACACAACCTAG
Ark2 RHR forward sequence	aacccggaattcgagctcggACAATTCATATAAAAGAAAGCG
Ark2 RHR reverse sequence	cgagagattgggtattagacctagggataacagggtaatCATGTATCATTACTACATAAACAAGA
Ark2 sgRNA target site	GGAGTTAATATTAAAAGTAA
PKAc RHR forward sequence	aacccggaattcgagctcggTGATTGGTAGAAAAATGAAGAGA
PKAc RHR reverse sequence	agagattgggtattagacctagggataacagggtaatTTTTTATTAAGGTAATACTTAACATATA ACATTTTC
PKAc sgRNA target site	GGTTCATTCGCATAAAAAGG

RHR, right homology region; cKD, conditional knockdown

Ark1 LHR and re-codonized region (944-1549 bp; stop codon removed)

CATTTGGATTTGAAACCCGAAAATGTTTTAGTAAACCATGAAGAAAAATGTAAGTTAGCTGATTTTGGTTTATC
AGCACATATAGGATCTAAACATAAAAAGAAAGGTATATCTCATTATAGAGGAACACATGATTATTGGTCTCCT
GAACAATGTGCAAGACATCAAAAGAAGAAACAGAATTTTGGAGAATTTGATCAGAAAACAGATATATGGACTC
TAGGGATTTTAGCTTTTGAATTAAAATTTGGTAGACCTCCATTTGGTTCAACAAATGAAGAAAGAGAAAATGTA
ATTATGAATAGAATACAAGATTATCATTGGAGTCAATTATTCTGTGAAAAAGTAAAACAAGATTTAATAGATAA
ATTATCACCTGAATTTAAAGATTTCTTAAATTTATGTCTAGATAAAAATCCAAAAAAAAGACCAACTGCAGAAT
CCTTAATTCAACATCCTTTCATCTTCATCCACAACAAGAACCGCTCCTGCATCAAGAGCTACGCCACCCAGCC
CCGCGGAAAGCAGGGCCCGAAGCTGAGCCACAAGGGCTTCAACGAGCAGGACGGCTCCTTCCTGACCCCC
ATCTGGTTCCACAATAAG

Ark2 LHR and re-codonized region (4596-5468 bp; stop codon removed)

ACTAGCCGACTTTGGATTTTCTTGTCAGCTCAAAAATAAAAGACAAAAAAGGAGTACATTCTGTGGCACAATC
GATTATATGCCCCCTGAAATTATTAACCAAATACCTTATGATTGTAATGTGGATCTGTGGTGTTTGGGTATAGT
TATATTTGAGTTGTTAGTTGGATTTCCTCCATTTACCGACGATACACAGgtaaaaaaataaaataaaaaaaaaaaaaattca
tatgtaattgaaatgaaataaaaacagtatgacatgtgcatataaaatggaataaaaaatatgacttgttcatgtaaaaaaaaaaaaaaaaaatatatatatata
tatatatatatatataaaaataacaaataaaataacatataaaattgctgacttgtacatgtaaaaaaaaaaaaaaaaaatatatatatatatatatatatataa
aaataacaaataaaataacatataaaattgctgacttgtacatgtgaaaactaccaattccaaaaagaacctttaaaaagacatttatgtaataataatatattattt
gatttattatatatgtatttttttttttttttttagGAGAGGATCTTTGATCAGATCAAGGAGCTGAACTTCCACTTCCCCAAGAGCGT
GAGCCTGCTGGCCCAGGAGCTGATTCTGAAGCTGTGCAGCCGCACCGCCGAGGAGCGCATCAGCGCCGAC
GAGGTGAAGAGCCACCCGTGGATCAAGCAGTTCATC

PKAc LHR and re-codonized region (975-1914 bp; stop codon removed)

tttacacatattcattcataattccacttcaactttataccttttttatttttgaagAGATTTGAAACCTGAAAATTTATTACTTGATAAAGATGG
ATTTATAAAAATGACTGATTTTGGATTTGCTAAAATAGTCGAGACGAGAACTTATACTTTATGTGGAACTCCAG
AATATATCGCTCCAGAAATTTTATTGAACGTCGGACATGGAAAAGCCgtatgcaaaaaaaaaaaatgtggaaaaattacatga
acatatgcatatttatattgaaaagtgtgaatttattacatgaacatgttcataatttttatgaaaaaatgtggactattacatgaacatgttcataaattttatgaaaaaa
tgtggactattacatgaacatgttcataatttttatgaaaaaatgtggattattacatgatcattttatatatatatatatatatatatatatatatgtatatatttttatatttat
atgtttatatcaataggtttatcaaagaaatataaaatttttatttttacattatagGCTGATTGGTGGACTTTAGGTATATTTATCTATGAGAT
CCTTGTGGGTTGCCCACCATTCTACGCTAACGAGCCACTTCTTATATACCAAAAGATCCTTGAGGGGATCAT
CTACTTTCCAAAGTTCCTTGACAATAACTGTAAGCACCTTATGAAAAAGCTTCTTAGCCATGACCTTACCAAG
CGTTACGGGAACCTCAAAAAGGGTGCACAGAACGTGAAGGAGCATCCTTGGTTCTCAAACATAGACTGGGT
TAACCTTCTTAACAAGAACGTGGAAGTGCCTTACAAGCCAAAGTACAAGAACATATTCGACAGTAGTAACTTC
GAACGTGTTCAGGAGGACCTTACGATCGCAGACAAGATCACCAACGAGAACGACCCTTTCTACGACTGG

3. Methods

3.1 Kinobeads screening

The assay including MS analysis was performed as described in Arendse *et al.*[60] Briefly chemoproteomic affinity capture experiments were performed as described previously[65] using Kinobeads,[66] which consist of promiscuous kinase inhibitors immobilized on Sepharose beads. Beads were equilibrated in lysis buffer and incubated at 4°C for 1 h with *P. falciparum* blood-stage protein extracts (*Pf*3D7 isolate; 0.3 mg), pretreated with either compound or DMSO (vehicle control). Protein depletion and IC_{50} values were calculated using a TMT 10-plex setup,[67] enabling simultaneous relative quantification across 10 conditions. Apparent dissociation constants (K_d^{app}) were determined by accounting for protein depletion during rebinding experiments (**Supplementary Table S1**). Proteins were processed using a modified SP3 protocol,[68] labeled with TMT10 reagents, and subjected to LC-MS/MS analysis on Q Exactive Orbitrap or Orbitrap Fusion Lumos mass spectrometers (Thermo Fisher Scientific.[65, 69] Protein identification was performed using Mascot 2.4 (Matrix Science) with <1% false discovery rate (FDR) for single-spectrum assignments and <0.1% FDR for multi-spectrum assignments. Quantified proteins required ≥2 unique peptide matches with <0.1% FDR. Raw data tables are available in the **Supplementary Table S1** MS Excel file.

3.2 *Plasmodium falciparum* parasitology methods

3.2.1. Parasite culture

Human malaria parasites were cultured as described previously with minor modifications.[70] Several culture-adapted strains of *P. falciparum* were used for the different assessments. Parasites were maintained at 5-10% parasitemia in human erythrocytes (type O+) suspended in RPMI-1640 (10.44 g/L) growth media supplemented with 25 mM HEPES, 4 g/L glucose, 0.088 g/L Hypoxanthine, 25 mM bicarbonate and 0.5% Albumax-II. Cultures were incubated at 37°C in a mixture of 3% O_2 and 4% CO_2 in nitrogen and had growth media replenished daily to ensure viability.

3.2.2 Antiplasmodial activity assessments

For the initial screen, the *in vitro* sensitivity of *Plasmodium falciparum* asexual blood stage parasites (3D7 strain) to the human kinase inhibitor set, including CHIR-124, was evaluated using a whole-cell [^{3}H]-hypoxanthine incorporation assay.[71] Compounds were tested in triplicate via a serial dilution series starting at 10 µM with a 1:3 dilution factor. Parasite growth inhibition was calculated relative to untreated controls, and IC_{50} values were determined by fitting dose-response curves. Each assay plate included appropriate growth, vehicle, and positive control wells.

For follow up studies, CHIR-124 was commercially obtained from Selleckchem (Catalog No.S2683, SMILES: C1CN2CCC1C(C2)NC3=C(C(=O)NC4=C3C=C(C=C4)Cl)C5=NC6=CC=CC=C6N5, CAS No. 405168-58-3). A full dose-response assessment was performed for CHIR-124 in a 96-well plate to determine the concentration inhibiting 50% of parasite growth (IC_{50} value), with parasite survival measured using lactate dehydrogenase (pLDH) activity.[72] Samples were prepared to a 10 mmol/L stock solution in 100% DMSO and stored at room temperature until testing. Dilutions to the desired starting concentration were freshly prepared in growth media on each occasion of the experiment The standard antimalarial drugs chloroquine and artesunate were used as the reference drugs in all experiments. The highest concentration of solvent to which the parasites were exposed was <0.5% and has no measurable effect on the parasite viability. The assay plate was incubated at 37 °C for 72 h in a sealed gas chamber under 3% O_2 and 4% CO_2 in nitrogen.

For activity assessed using the pLDH method, the wells in the assay plate were gently resuspended after 72 h, and 15 µL from each well was transferred to a corresponding well in a duplicate plate containing 100 µL of Malstat reagent and 25 µL of nitroblue tetrazolium solution. Plates were left to develop for 20 minutes in the dark and then absorbance of each well was quantified using a spectrophotometer at 620 nm wavelength. Regression analysis was performed using the Dotmatics software platform to quantify the IC_{50} value.

3.2.3. *Stage specificity*

Stage specificity assessments were carried out as described previously, using the pLDH assay as above with minor modifications.[73] Parasites at each stage were exposed to a range of drug concentrations from 6 µmol/L to 12 nmol/L and growth at each concentration compared between stages. Assessment of ring stage activity was carried out using a synchronous ring-stage culture at 2% parasitemia. Growth was determined via pLDH activity after 24h in the late trophozoite stage when pLDH activity is greatest. Schizont stage assessments were carried out using synchronous schizont cultures, and growth was determined after 48h in the next late trophozoite stage. Comparison of the relative parasite survival at each concentration between the two stages showed when the killing action was taking place.

3.2.4 *Stage-specific gametocyte production and luciferase assay*

Gametocytogenesis was induced from a tightly synchronised (>97 % rings, 0.5 % parasitemia, 6 % hematocrit grown in RPMI media containing Albumax II in A+/O+ human erythrocytes) asexual parasite culture from a NF54-Pfs16-GFP-luc *P. falciparum* transgenic line, under stationary, hypoxic conditions as described before.[74] The hematocrit was reduced to 4 % after on day 0 as an anemic stressor and immature gametocytes (stage II/III) were harvested on day 5-6 from cultures exposed to 50 mM N-acetyl glucosamine (NAG) on days 1-4. For late-stage (stage IV/V) and/or mature (stage V) gametocytes, NAG treatment occurred from days 3-7, and gametocytes were harvested on day 10 or 13.

Compounds were evaluated for stage-specific gametocytocidal activity with a 48 h drug pressure on either immature (stage II/III) or mature (stage V) gametocytes (2 % gametocytemia, 1.5 % hematocrit) under hypoxic conditions at 37 °C. Luciferase activity was determined as a proxy for viability as described before.[74] Effective half-maximal inhibition (IC_{50}) values were determined for 2-fold dilutions over 9 concentration points, in technical triplicates and for three independent biological replicates, with methylene blue and MMV390048 serving as internal controls. IC_{50} values were determined through non-linear, 4-parameter curve fitting in GraphPad and are indicated as means ± S.E.

3.2.5 *Liver-stage assay*

Plasmodium berghei (Pb) sporozoites were obtained by dissecting the salivary glands of infected *Anopheles stephensi* mosquitoes. These were provided by The SporoCore, at the University of Georgia, GA, USA (SporoCore.uga.edu). The parasites utilized a GFP-$Luc_{ama1\text{-}eef1}$ reporter line.[75] These engineered parasites, termed Pb-Luc, were used to infect HepG2-A16-CD81EGFP cells.

HepG2-A16-CD81EGFP cells stably transformed to express a GFP-CD81 fusion protein[76] were cultured at 37°C in 5% CO_2 in DMEM (Invitrogen, Carlsbad, USA) supplemented with 10% FBS (Corning, NY, USA), 1X Pen/Strep/Glu (Thermo Fisher Scientific, USA).

Compounds, including CHIR-124 (Selleckchem, Catalog No.S2683) and controls were prepared as 10 mM solutions in DMSO, and 10 nl of each (resulting in a final DMSO concentration of 0.1% per well) was transferred into assay plates using an ECHO 650 (Beckman). Concentrations ranged from 10 µM to 0.5 nM. Atovaquone (1 µM) served as a positive control, while 0.1% DMSO was used as a negative control. Human hepatic cells (HepG2-A16-CD81-EGFP) suspended in 5 µL of DMEM medium ($2x10^5$ cells/ml, supplemented with 5%FBS, 5XPen/Strep/Glu) were seeded (at 3×10^3 per well) in 1536-well plates (Greiner BioOne) 20 hours prior to infection. PbLuc sporozoites, isolated from the salivary glands of *A. stephensi* mosquitoes were filtered twice through a 20 µm nylon pore cell strainer. The sporozoites were resuspended in screening media, counted with a hemocytometer, and diluted to a final concentration of 200 sporozoites per µL. Each well received 1,000 sporozoites in 5 µL, and the plates were centrifuged for 3 minutes in an Eppendorf 5810 R (330 RCF) on the lowest acceleration and brake settings. After incubation at 37°C in 5% CO_2 for 48 hours, media was removed by centrifuging the inverted plates at 130 RCF for 1 minute. Wells then received 2 µL of Bright-Glo™ Luciferase Assay System (Promega).[77] Luminescence was measured immediately using a Pherastar FSX reader (BMG Labtech). Luminescence values were normalized using the positive and negative controls. EC_{50} values were calculated with CDD Vault (Burlingame, CA). All experiments were performed with at least four technical replicates and repeated three times.

3.2.6 *Liver cytotoxicity assay*

Plates were prepared following the liver stage conditions, except that 5 µl of the media was used instead of sporozoites.[77] After incubation at 37°C for 48 hours, media was removed by spinning the inverted plates at 130 RCF for 1 minute, after which 2 µl of CellTiter-Glo® Luminescent Cell Viability Assay (Promega) was dispensed in each well. Immediately after adding the CellTiter-Glo reagent, the plates were read by the Pherastar FSX reader (BMG Labtech). Luminescence intensity values were normalized against positive (Puromycin) and negative (DMSO) controls. EC_{50} values were calculated in CDD Vault (Burlingame, CA). All experiments were performed in four technical replicates and repeated at least three times.

3.2.7 P. falciparum *conditional knockdown assays*

The NF54 strain and PDK1 cKD parasites were cultured in the presence (control conditions) or absence of 675 nM Shield-1 (PfPDK1 knockdown conditions) as described by Hitz et al.[61] *In vitro* IC_{50} determination of CHIR-124 against *P. falciparum* asexual blood stage (PDK1 cKD or control) parasites βwas performed with a modified [^{3}H]-hypoxanthine incorporation assay, as previously reported.[78]

Ark1 (PF3D7_0605300), Ark2 (PF3D7_0309200), and PKAc (PF3D7_0934800) cKD lines generated by fusing the coding sequence and non-coding RNA aptamer sequences in the 5'- and 3'-UTR, permitting translation regulation using the TetR-DOZI system.[79] Editing was achieved by CRISPR/SpCas9 using the linear pSN054 vector that contains cloning sites for the left homology region (LHR) and the right homology region (RHR) as well a gene-specific guide RNA under control of the T7 promoter. Constructs were clone into the pSN054 donor vector[80] that includes preinstalled V5-2xHA epitope tags, a 10x tandem array of TetR aptamers, and a multicistronic cassette for expression of TetR-DOZI (regulation), blasticidin S-deaminase (selection marker) and a Renilla luciferase (RLuc) reporter. The LHR and recoded region was installed in-frame with tandem V5-2x-hemagglutinin (HA) tags to afford C-terminal epitope-tagged proteins post-editing, and upstream of the regulatory aptamer array. All primer and synthetic fragment sequences generated using the BioXP™ system are included in **Supplementary Table S6**. The final construct was sequence-verified and further confirmed by restriction digests. Transfection into Cas9- and T7 RNA polymerase-expressing NF54 parasites was carried out by pre-loading erythrocytes with the donor vector as previously described.[81] Parasite culture was maintained continuously in 500 nM anhydrotetracycline (aTc, Sigma-Aldrich 37919) and drug selection with 2.5 µg/mL of Blasticidin S (RPI Corp B12150-0.1) was initiated four days after transfection. Cultures were monitored by Giemsa smears and RLuc measurements.

Compound susceptibility assays using *P. falciparum* Ark1, Ark2, PKAc and PI4K cKD lines were carried out as previously described.[79, 80] Briefly, synchronous ring-stage Ark1 (PF3D7_0605300), Ark2 (PF3D7_0309200), PKAc (PF3D7_0934800) and PI4K (PF3D7_0509800) cKD parasites, as well as a control parasite line expressing an aptamer-regulatable fluorescent protein were maintained in the presence of high aTc (500 nM) or no aTc and distributed into 384-well polystyrene microplates (Corning). Stock solutions of compounds were serially diluted and transferred to the parasite-containing plates using the Janus platform (PerkinElmer). DMSO and dihydroartemisinin treatment (500 nM) served as reference controls. Luminescence was measured after 72 h using the Renilla-Glo Luciferase Assay System (Promega E2750) and the GloMax Discover Multimode Microplate Reader (Promega), and IC_{50} values were obtained from corrected dose-response curves using Graph-Pad Prism.

For the generation of the GSK3β cKD lines, parasite culture medium excluded glucose, B+ human erythrocytes were used and cultures were incubated with 1% O_2 and 5% CO_2. The lines were generated by selection linked integration (SLI) as described previously.[82] GSK3β-GFP-glmS[83] was generated by endogenous C-terminal GFP-tagging of the target gene thereby introducing a glmS ribozyme sequence[84] upstream of the 3' untranslated region as described previously.[63] GSK3β-DiCre was generated using a N-terminal homology region of the target gene followed by a recodonized version of the C-terminal kinase domain flanked by artificial introns containing loxP sites as described previously.[64] Subsequently, parasites were transfected with an episomal copy of a dimerizable Cre recombinase (DiCre) and excision of the GSK3β kinase domain was induced by rapalog-mediated dimerization of DiCre as described previously.[64]

Drug sensitivity assays with GSK3β-cKD lines were carried out as described previously).[85] Briefly, synchronous ring stage parasites were seeded in to black flat-bottom 96 well plates (ThermoFisher) at 0.1 % parasitemia and 2% hematocrit at a final volume of 200 µl. GlmS-based knock down and DiCre-based knockout were induced by treatment with 2.5 mM Glucosamine or 250 nM rapalog (Takara Clontech) respectively. Vehicle treated parasites served as a control. Compound at varying concentrations

was added in triplicates and parasites were incubated for 96 h under standard culture conditions. Parasite proliferation was quantified based on the DNA content by replacing 100 µl of the supernatant with 1x SYBR Gold stain (Invitrogen) in lysis buffer (20 mM Tris-HCl pH 7.5; 5 mM EDTA, 0.008% saponin; 0.08% Triton-X-100). Fluorescence of SYBR-Gold-labelled DNA was quantified using an EnVision multilabel plate reader (PerkinElmer, 485 nm excitation, 535 nm emission) as described previously.[86] IC_{50} values were calculated from dose-response curves using GraphPad Prism.

3.2.8 Cross-resistance profiling using the Antimalarial Resistome Barcode sequencing assay (AReBar)

Profiling of cross-resistance using a pooled library of barcoded mutant parasite lines was performed as previously described.[87, 88] The pool consisted of parasites in the *Pf*3D7 and *Pf*Dd2 backgrounds, with mutations described in **Supplementary Table S4**. The pool was exposed to either 3×IC_{50} of CHIR-124, or untreated (control), and cultured for 14 days with growth measured by flow cytometry every 2-3 days. Parasitemia was maintained below 5%. PCR-amplified barcodes were quantified by next generation sequencing using an Oxford Nanopore minION. The log2 fold change (LFC) in proportion was calculated relative to the untreated control pool, with a LFC >2.5 indicating cross-resistance.

3.2.9 Combination studies via fixed-ratio isobolograms

Isobolograms were determined using the fixed-ratio isobole analysis method previously described.[89] This was carried out by treating intraerythrocytic ABS parasites with fixed drug combination ratios of CHIR-124 and chloroquine or hesperadin set at 5:0, 4:1, 3:2, 2:3, 1:4, and 0:5. Independent dose-response curves of the fixed drug combinations were determined using the pLDH assay against NF54 ABS parasites for 72 h. Fractional IC_{50} values were calculated individually for each drug by plotting the relevant fractional concentration vs % parasite survival using GraphPad Prism v10. Mean fractional IC_{50} values for each drug pair were plotted and summed to determine effects of synergy, additivity or antagonism.

3.2.10 Rate- and stage-specific morphological evaluations and inhibitor effect reversibility

To determine stage specificity during asexual intra-erythrocytic development, *P. falciparum* NF54 parasites were synchronised using 5 % D-Sorbitol to obtain a >90 % ring-stage population (6 – 10 h post-invasion, hpi). *In vitro*, synchronised parasite cultures were treated with CHIR-124 (3×IC_{50}). The effect of CHIR-124 on various asexual intra-erythrocytic stage parasites (ring, early/late trophozoite, and schizont) was monitored morphologically every 12 h using Giemsa-stained thin smears. Images were captured using a Nikon Eclipse 50i light microscope adapted with a Nikon camera (Nikon DS-Fi1) and NIS-Elements software.

To further assess and quantify the specific parasite stage at which CHIR-124 is active and to determine whether the effect is reversible, as described above, various asexual intra-erythrocytic stages were treated with CHIR-124 at 3×IC_{50} for 12-h segments. After which, CHIR-124 was washed off, and parasites were left to recover in drug-free medium. Parasitaemia and nuclear content were monitored every 12 h until the next ABS life cycle using flow cytometry, as described in *van Biljon R et al.*[90]

3.3. β-hematin/hemozoin formation inhibition assays and metal chelation studies

3.3.1 NP-40 based extracellular β-hematin formation inhibition assay

The NP-40 detergent based assay methods for inhibitors of βH formation described by Sandlin *et al.*[91] were modified for manual liquid delivery. Samples were dissolved in DMSO to give 10 mM solutions and 20 µL of each was delivered to wells in the last column (column 12) of a 96-well plate together with distilled water (140 µL) and NP40 detergent (305.5 mM, 40 µL). A solution containing water/NP40 (305.5 mM)/DMSO at a v/v ratio of 70%/20%/10% respectively was prepared and then 100 µL was added to all other wells (columns 1–11). A serial dilution of each compound (100 µL) from column 12 down to column 2 was carried out. Column 1 served as a blank with 0 mM sample. A 25 mM hematin stock solution was prepared by sonicating hemin in DMSO for one minute and then suspending 178 µL of this in a 1 M acetate buffer (20 mL, pH 4.8). The homogenous suspension (100 µL) was then added to the wells to give final buffer and haematin concentrations of 0.5 M and 100 µM respectively. The plate was covered and incubated at 37 °C for 5–6 h in an incubator. Analysis was carried out using the pyridine-ferrichrome method developed by Ncokazi and Egan.[92] A solution of 50% (v/v) pyridine, 30% (v/v) H_2O, 20% (v/v)

acetone and 0.2 M HEPES buffer (pH 7.4) was prepared and 32 µL added to each well to give a final pyridine concentration of 5% (v/v). Acetone (60 µL) was then added to assist with hematin dispersion. The UV-vis absorbance of the plate wells was read on a Thermo Scientific MultiskanGO plate reader. Sigmoidal dose-response curves were fitted to the absorbance data using GraphPad Prism v5 to obtain a 50% inhibitory concentration (IC_{50}) for each compound.

3.3.2 Cellular heme fractionation assay

Heme fractionation analysis was performed as described.[93] Briefly, early ring-stage sorbitol- synchronized NF54 parasites were exposed to CHIR-124 at concentrations corresponding to 0.5, 1, 1.5, 2 and 3 times the ABS IC_{50}. After a 32 h incubation, cells were saponin-lysed and trophozoites were subjected to a series of lysis, solubilization, and differential centrifugation steps to obtain fractions that contain Hb, free heme or Hz. The UV-visible spectra of heme as Fe(III)heme-pyridine was analyzed in each of these fractions and compared to a heme standard curve for accurate quantification. These quantities were subsequently normalized using the number of analyzed cells, as determined by flow cytometry of an aliquot of exposed cells. Two-tailed t-tests were used to assess significance.

3.3.3 Metal chelation studies

To construct Job plots to determine, qualitatively, the extent of chelation between CHIR-124 and physiologically-relevant metal ions, the absorption spectra of solutions of varying mole fractions of CHIR-124 and metal ions were recorded according to a method previously described, with modifications.[94] Briefly, the total concentration of the system was constrained to 0.3 mM and the absorbance was measured from 200 nm to 600 nm in a 1.00 mm path length cuvette in 20 mM PBS at pH 7.4. Working solutions with mole fractions of metal ion between 0 and 1 were prepared, i.e. at metal ion mole fractions of 0, 0.2, 0.4, 0.6, 0.8 and 1, with the remainder of the mole fraction provided by a complementary amount of CHIR-124 (1, 0.8, 0.6, 0.4, 0.2 and 0, respectively). Spectrophotometric data were analysed at the absorbance maximum of CHIR-124 (364 nm) and normalised according the maximum and minimum absorbance values recorded for each experiment.

3.4 Biochemical assays

3.4.1 KinaseSeeker assay

KinaseSeeker is a homogeneous competition binding assay where the displacement of an active site-dependent probe by an inhibitor is measured by a change in luminescence signal. Luminescence readout translates into a highly sensitive and robust assay with low background and minimal interference from test compounds.[95, 96]

10 mM stock solutions of compounds were serially diluted to obtain assay stocks. Prior to initiating a profiling campaign, the compounds were evaluated for false positive against split-luciferase. The compounds were then screened in duplicate against each of the kinases. For kinase assays, each Cfluc-Kinase was translated along with Fos-Nfluc using a cell-free system (rabbit reticulocyte lysate) at 30 ˚C for 90 min. 24 µL aliquot of this lysate containing either 1 µL of DMSO (for no-inhibitor control) or compound solution in DMSO (various concentrations) was incubated for 2 hours at room temperature in presence of a kinase specific probe. 80 µL of luciferin assay reagent was added to each solution and luminescence was measured on a luminometer.

The % activity remaining was calculated using the following equation:

$$\%\ \text{Inhibition} = \frac{\text{ALU}_{\text{Control}} - \text{ALU}_{\text{Sample}}}{\text{ALU}_{\text{Control}}} \times 100$$

$$\%\ \text{Activity Remaining} = 100 - \%\ \text{Inhibition}$$

References

(60) Arendse, L. B.; Murithi, J. M.; Qahash, T.; Pasaje, C. F. A.; Godoy, L. C.; Dey, S.; Gibhard, L.; Ghidelli-Disse, S.; Drewes, G.; Bantscheff, M.; et al. The anticancer human mTOR inhibitor sapanisertib potently inhibits multiple *Plasmodium* kinases and life cycle stages. *Sci Transl Med* **2022**, *14* (667), eabo7219. DOI: 10.1126/scitranslmed.abo7219.
(61) Hitz, E.; Wiedemar, N.; Passecker, A.; Graca, B. A. S.; Scheurer, C.; Wittlin, S.; Brancucci, N. M. B.; Vakonakis, I.; Maser, P.; Voss, T. S. The 3-phosphoinositide-dependent protein kinase 1 is an essential upstream activator of protein kinase A in malaria parasites. *PLoS Biol* **2021**, *19* (12), e3001483. DOI: 10.1371/journal.pbio.3001483.
(62) Goldfless, S. J.; Wagner, J. C.; Niles, J. C. Versatile control of *Plasmodium falciparum* gene expression with an inducible protein-RNA interaction. *Nat Commun* **2014**, *5*, 5329. DOI: 10.1038/ncomms6329.
(63) Burda, P. C.; Crosskey, T.; Lauk, K.; Zurborg, A.; Sohnchen, C.; Liffner, B.; Wilcke, L.; Pietsch, E.; Strauss, J.; Jeffries, C. M.; et al. Structure-Based Identification and Functional Characterization of a Lipocalin in the Malaria Parasite *Plasmodium falciparum*. *Cell Rep* **2020**, *31* (12), 107817. DOI: 10.1016/j.celrep.2020.107817 From NLM Medline.
(64) Jones, M. L.; Das, S.; Belda, H.; Collins, C. R.; Blackman, M. J.; Treeck, M. A versatile strategy for rapid conditional genome engineering using loxP sites in a small synthetic intron in *Plasmodium falciparum*. *Sci Rep* **2016**, *6*, 21800. DOI: 10.1038/srep21800.
(65) Balestra, A. C.; Koussis, K.; Klages, N.; Howell, S. A.; Flynn, H. R.; Bantscheff, M.; Pasquarello, C.; Perrin, A. J.; Brusini, L.; Arboit, P.; et al. Ca(2+) signals critical for egress and gametogenesis in malaria parasites depend on a multipass membrane protein that interacts with PKG. *Sci Adv* **2021**, *7* (13). DOI: 10.1126/sciadv.abe5396.
(66) Bantscheff, M.; Hopf, C.; Savitski, M. M.; Dittmann, A.; Grandi, P.; Michon, A. M.; Schlegl, J.; Abraham, Y.; Becher, I.; Bergamini, G.; et al. Chemoproteomics profiling of HDAC inhibitors reveals selective targeting of HDAC complexes. *Nat Biotechnol* **2011**, *29* (3), 255–265. DOI: 10.1038/nbt.1759.
(67) Werner, T.; Sweetman, G.; Savitski, M. F.; Mathieson, T.; Bantscheff, M.; Savitski, M. M. Ion coalescence of neutron encoded TMT 10-plex reporter ions. *Anal Chem* **2014**, *86* (7), 3594–3601. DOI: 10.1021/ac500140s.
(68) Moggridge, S.; Sorensen, P. H.; Morin, G. B.; Hughes, C. S. Extending the Compatibility of the SP3 Paramagnetic Bead Processing Approach for Proteomics. *J Proteome Res* **2018**, *17* (4), 1730–1740. DOI: 10.1021/acs.jproteome.7b00913.
(69) Sridharan, S.; Kurzawa, N.; Werner, T.; Gunthner, I.; Helm, D.; Huber, W.; Bantscheff, M.; Savitski, M. M. Proteome-wide solubility and thermal stability profiling reveals distinct regulatory roles for ATP. *Nat Commun* **2019**, *10* (1), 1155. DOI: 10.1038/s41467-019-09107-y.
(70) Trager, W.; Jensen, J. B. Human malaria parasites in continuous culture. *Science* **1976**, *193* (4254), 673–675. DOI: 10.1126/science.781840.
(71) Mandt, R. E. K.; Lafuente-Monasterio, M. J.; Sakata-Kato, T.; Luth, M. R.; Segura, D.; Pablos-Tanarro, A.; Viera, S.; Magan, N.; Ottilie, S.; Winzeler, E. A.; et al. In vitro selection predicts malaria parasite resistance to dihydroorotate dehydrogenase inhibitors in a mouse infection model. *Sci Transl Med* **2019**, *11* (521). DOI: 10.1126/scitranslmed.aav1636 From NLM Medline.
(72) Makler, M. T.; Ries, J. M.; Williams, J. A.; Bancroft, J. E.; Piper, R. C.; Gibbins, B. L.; Hinrichs, D. J. Parasite lactate dehydrogenase as an assay for *Plasmodium falciparum* drug sensitivity. *Am J Trop Med Hyg* **1993**, *48* (6), 739–741. DOI: 10.4269/ajtmh.1993.48.739.
(73) Le Manach, C.; Scheurer, C.; Sax, S.; Schleiferbock, S.; Cabrera, D. G.; Younis, Y.; Paquet, T.; Street, L.; Smith, P.; Ding, X. C.; et al. Fast in vitro methods to determine the speed of action and the stage-specificity of anti-malarials in *Plasmodium falciparum*. *Malar J* **2013**, *12*, 424. DOI: 10.1186/1475-2875-12-424.
(74) Reader, J.; van der Watt, M. E.; Birkholtz, L. M. Streamlined and Robust Stage-Specific Profiling of Gametocytocidal Compounds Against *Plasmodium falciparum*. *Front Cell Infect Microbiol* **2022**, *12*, 926460. DOI: 10.3389/fcimb.2022.926460.
(75) Pathak, A. K.; Shiau, J. C.; Franke-Fayard, B.; Shollenberger, L. M.; Harn, D. A.; Kyle, D. E.; Murdock, C. C. Streamlining sporozoite isolation from mosquitoes by leveraging the dynamics of migration to the salivary glands. *Malar J* **2022**, *21* (1), 264. DOI: 10.1186/s12936-022-04270-y.
(76) Yalaoui, S.; Zougbede, S.; Charrin, S.; Silvie, O.; Arduise, C.; Farhati, K.; Boucheix, C.; Mazier, D.; Rubinstein, E.; Froissard, P. Hepatocyte permissiveness to *Plasmodium* infection is conveyed by a short and structurally conserved region of the CD81 large extracellular domain. *PLoS Pathog* **2008**, *4* (2), e1000010. DOI: 10.1371/journal.ppat.1000010.
(77) Swann, J.; Corey, V.; Scherer, C. A.; Kato, N.; Comer, E.; Maetani, M.; Antonova-Koch, Y.; Reimer, C.; Gagaring, K.; Ibanez, M.; et al. High-Throughput Luciferase-Based Assay for the Discovery of Therapeutics That Prevent Malaria. *ACS Infect Dis* **2016**, *2* (4), 281–293. DOI: 10.1021/acsinfecdis.5b00143.
(78) Snyder, C.; Chollet, J.; Santo-Tomas, J.; Scheurer, C.; Wittlin, S. In vitro and in vivo interaction of synthetic peroxide RBx11160 (OZ277) with piperaquine in *Plasmodium* models. *Exp Parasitol* **2007**, *115* (3), 296–300. DOI: 10.1016/j.exppara.2006.09.016.
(79) Ganesan, S. M.; Falla, A.; Goldfless, S. J.; Nasamu, A. S.; Niles, J. C. Synthetic RNA-protein modules integrated with native translation mechanisms to control gene expression in malaria parasites. *Nat Commun* **2016**, *7*, 10727. DOI: 10.1038/ncomms10727.
(80) Nasamu, A. S.; Falla, A.; Pasaje, C. F. A.; Wall, B. A.; Wagner, J. C.; Ganesan, S. M.; Goldfless, S. J.; Niles, J. C. An integrated platform for genome engineering and gene expression perturbation in *Plasmodium falciparum*. *Sci Rep* **2021**, *11* (1), 342. DOI: 10.1038/s41598-020-77644-4.
(81) Deitsch, K.; Driskill, C.; Wellems, T. Transformation of malaria parasites by the spontaneous uptake and expression of DNA from human erythrocytes. *Nucleic Acids Res* **2001**, *29* (3), 850–853. DOI: 10.1093/nar/29.3.850.
(82) Birnbaum, J.; Flemming, S.; Reichard, N.; Soares, A. B.; Mesen-Ramirez, P.; Jonscher, E.; Bergmann, B.; Spielmann, T. A genetic system to study *Plasmodium falciparum* protein function. *Nat Methods* **2017**, *14* (4), 450–456. DOI: 10.1038/nmeth.4223.
(83) Alder, A.; Wilcke, L.; Pietsch, E.; von Thien, H.; Pazicky, S.; Low, C.; Mesen-Ramirez, P.; Bachmann, A.; Burda, P. C.; Kunick, C.; et al. Functional inactivation of *Plasmodium falciparum* glycogen synthase kinase GSK3 modulates erythrocyte invasion and blocks gametocyte maturation. *J Biol Chem* **2022**, *298* (9), 102360. DOI: 10.1016/j.jbc.2022.102360.

(84) Prommana, P.; Uthaipibull, C.; Wongsombat, C.; Kamchonwongpaisan, S.; Yuthavong, Y.; Knuepfer, E.; Holder, A. A.; Shaw, P. J. Inducible knockdown of *Plasmodium* gene expression using the glmS ribozyme. *PLoS One* **2013**, *8* (8), e73783. DOI: 10.1371/journal.pone.0073783.
(85) Alder, A.; Sanchez, C. P.; Russell, M. R. G.; Collinson, L. M.; Lanzer, M.; Blackman, M. J.; Gilberger, T. W.; Matz, J. M. The role of *Plasmodium* V-ATPase in vacuolar physiology and antimalarial drug uptake. *Proc Natl Acad Sci U S A* **2023**, *120* (30), e2306420120. DOI: 10.1073/pnas.2306420120.
(86) Smilkstein, M.; Sriwilaijaroen, N.; Kelly, J. X.; Wilairat, P.; Riscoe, M. Simple and inexpensive fluorescence-based technique for high-throughput antimalarial drug screening. *Antimicrob Agents Chemother* **2004**, *48* (5), 1803–1806. DOI: 10.1128/AAC.48.5.1803-1806.2004 .
(87) Carrasquilla, M.; Drammeh, N. F.; Rawat, M.; Sanderson, T.; Zenonos, Z.; Rayner, J. C.; Lee, M. C. S. Barcoding Genetically Distinct *Plasmodium falciparum* Strains for Comparative Assessment of Fitness and Antimalarial Drug Resistance. *mBio* **2022**, *13* (5), e0093722. DOI: 10.1128/mbio.00937-22.
(88) Mambwe, D.; Coertzen, D.; Leshabane, M.; Mulubwa, M.; Njoroge, M.; Gibhard, L.; Girling, G.; Wicht, K. J.; Lee, M. C. S.; Wittlin, S.; et al. hERG, *Plasmodium* Life Cycle, and Cross Resistance Profiling of New Azabenzimidazole Analogues of Astemizole. *ACS Med Chem Lett* **2024**, *15* (4), 463–469. DOI: 10.1021/acsmedchemlett.3c00496.
(89) Fivelman, Q. L.; Adagu, I. S.; Warhurst, D. C. Modified fixed-ratio isobologram method for studying in vitro interactions between atovaquone and proguanil or dihydroartemisinin against drug-resistant strains of *Plasmodium falciparum*. *Antimicrob Agents Chemother* **2004**, *48* (11), 4097–4102. DOI: 10.1128/AAC.48.11.4097-4102.2004.
(90) van Biljon, R.; Niemand, J.; van Wyk, R.; Clark, K.; Verlinden, B.; Abrie, C.; von Gruning, H.; Smidt, W.; Smit, A.; Reader, J.; et al. Inducing controlled cell cycle arrest and re-entry during asexual proliferation of *Plasmodium falciparum* malaria parasites. *Sci Rep* **2018**, *8* (1), 16581. DOI: 10.1038/s41598-018-34964-w.
(91) Sandlin, R. D.; Carter, M. D.; Lee, P. J.; Auschwitz, J. M.; Leed, S. E.; Johnson, J. D.; Wright, D. W. Use of the NP-40 detergent-mediated assay in discovery of inhibitors of beta-hematin crystallization. *Antimicrob Agents Chemother* **2011**, *55* (7), 3363–3369. DOI: 10.1128/AAC.00121-11.
(92) Ncokazi, K. K.; Egan, T. J. A colorimetric high-throughput beta-hematin inhibition screening assay for use in the search for antimalarial compounds. *Anal Biochem* **2005**, *338* (2), 306–319. DOI: 10.1016/j.ab.2004.11.022.
(93) Combrinck, J. M.; Fong, K. Y.; Gibhard, L.; Smith, P. J.; Wright, D. W.; Egan, T. J. Optimization of a multi-well colorimetric assay to determine haem species in *Plasmodium falciparum* in the presence of anti-malarials. *Malar J* **2015**, *14*, 253. DOI: 10.1186/s12936-015-0729-9.
(94) Woodland, J. G.; Hunter, R.; Smith, P. J.; Egan, T. J. Shining new light on ancient drugs: preparation and subcellular localisation of novel fluorescent analogues of Cinchona alkaloids in intraerythrocytic *Plasmodium falciparum*. *Org Biomol Chem* **2017**, *15* (3), 589–597. DOI: 10.1039/c6ob02110g.
(95) Jester, B. W.; Cox, K. J.; Gaj, A.; Shomin, C. D.; Porter, J. R.; Ghosh, I. A coiled-coil enabled split-luciferase three-hybrid system: applied toward profiling inhibitors of protein kinases. *J Am Chem Soc* **2010**, *132* (33), 11727–11735. DOI: 10.1021/ja104491h.
(96) Jester, B. W.; Gaj, A.; Shomin, C. D.; Cox, K. J.; Ghosh, I. Testing the promiscuity of commercial kinase inhibitors against the AGC kinase group using a split-luciferase screen. *J Med Chem* **2012**, *55* (4), 1526–1537. DOI: 10.1021/jm201265f.
